# Comparison Between Complications of Elective and Emergency Tracheostomies

**DOI:** 10.7759/cureus.48612

**Published:** 2023-11-10

**Authors:** Naeema Natasha, Saleh Khurshied, Muhammad A Zahid, Nawal Khurshid, Altaf Hussain, Muhammad A Khizer, Maesum Ali

**Affiliations:** 1 Otolaryngology - Head and Neck Surgery, Pakistan Institute of Medical Science, Islamabad, PAK; 2 Otolaryngology - Head and Neck Surgery, Pakistan Institute of Medical Sciences, Islamabad, PAK; 3 Ophthalmology, Monash Health, Clayton, AUS; 4 Ophthalmology, National University of Medical Sciences, Rawalpindi, PAK; 5 Ophthalmology, Armed Forces Institute of Ophthalmology, Rawalpindi, PAK

**Keywords:** icu( intensive care unit ), otolaryngology head and neck, emergency and elective surgery, post op complication, tracheostomy

## Abstract

Introduction

Tracheostomy, although a common surgical procedure, is associated with potential complications. Complications can be avoided with accurate technique and proper operative and postoperative care. A surgeon should know the complications of the procedure and how to avoid them so that complications may be prevented or minimized.

Objective

To determine the frequency of complications in patients undergoing elective and emergency tracheostomies.

Methods

The cross-sectional study was conducted at the Department of Otorhinolaryngology - Head and Neck Surgery, and Intensive Care Unit (ICU) of PIMS Hospital, Islamabad, from March 1 to August 31, 2023, with a total duration of six months. A total of 110 patients admitted to the ICU and presented in an emergency falling within the inclusion criteria were counted in the study. Patients were split into two groups, i.e., elective tracheostomy (group A) and emergency tracheostomy (group B). All patients were followed for three months for adverse events related to the tracheostomy. All information was collected on Proforma and analyzed using the SPSS 23 version (IBM Corp., Armonk, NY).

Results

The mean age of participants was 48.47±12.68 years in group A and 49.54±10.99 years in group B (p=0.636). 40 (72.7%) and 37 (67.3%) patients were male and 15 (27.3%) and 18 (32.7%) female in groups A and B, respectively. The results of post-operative complications in both groups A and B were observed, respectively, for surgical emphysema (2 [3.6%] vs. 5 [9.1%], p=0.241), hemorrhage (2 [3.6%] vs. 4 [7.3%], p=0.401), wound infection (3 [3.6%] vs. 6 [10.9%], p=0.279), tube blockage (0 [0.0%] vs. 1 [1.8%], p=0.315), tube displacement (0 [0.0%] vs. 3 [5.5%], p=0.079), and tracheoesophageal fistula (0 [0.0%] vs. 2 [3.6%], p=0.154). The overall complications in elective tracheostomies were 7 (12.7%) and in emergency tracheostomies were 21 (38.2%).

Conclusion

We concluded that patients who had an emergency tracheostomy experienced more postoperative complications than those who underwent an elective tracheostomy.

## Introduction

Emergency management of the airway has always been a challenging task for clinicians [1]. Tracheostomy is a common procedure in which a stoma (opening) is created in the skin in front of the neck, which communicates with the trachea [2].

Tracheostomy can also be done electively under general anesthesia in ICU patients who need prolonged mechanical intubation for cleaning of tracheobronchial secretions, protection of the lower respiratory tract, and to avoid the complications associated with tracheal intubation, e.g., subglottic stenosis [3]. A tracheostomy provides a low-resistance path for the exchange of air in the case of upper respiratory tract obstruction [4].

A tracheostomy is also recommended by many professional organizations in non-traumatic conditions with ineffective ventilator weaning at post-endotracheal tube intubation days 5-7. When trauma to the head, neck, or upper aerodigestive tumors ensues, a prophylactic tracheostomy may be required. As a result of the surgery or radiation therapy, there is a risk that the upper airway will become blocked, and tracheostomies are recommended before starting treatment [5].

No doubt there is a high chance of complications in tracheostomy, but only a few complications are serious, which can lead to morbidity and mortality. Healthcare providers should always be prepared to deal with these situations, which commonly include hemorrhage, dislodgement, and obstruction, while others less common include tracheoesophageal fistula, tracheocutaneous fistula, infection, and tracheal stenosis [6].

Doctors and nurses should take adequate measures to prevent complications by ensuring the early mobilization and suctioning of secretions regularly. The patient’s bedside should be equipped with a manual resuscitation bag, oxygen source, and tracheostomy kit [7].

Tracheostomy is no doubt a common lifesaving procedure but is associated with complications, which can be avoided with adequate technique and accurate operative and postoperative care. As there are limited data available in our country for tracheostomy complications and their solutions, the rationale of this study is to have a closer look at the complications in both types of tracheostomy so that they can be prevented and treated accordingly.

## Materials and methods

This study was carried out in the Department of Otorhinolaryngology and Head Neck Surgery and Intensive Care Unit of Pakistan Institute of Medical Sciences (PIMS) Islamabad, a tertiary health care hospital in Islamabad, Pakistan, after taking permission from the Institutional Ethical Clearance Committee (Ref No. F.1-1/2015/ERB/SZAMBU/1085). This study was carried out from March 1 to August 31, 2023, over a period of six months.

The sample size was calculated by using the WHO sample size calculator for two groups, taking the level of significance as 5%, the power of the test as 90%, the anticipated population proportion of elective tracheostomy patients (group A) = 9.99% [8], and the anticipated population proportion of emergency tracheotomy patients (group B) = 33.35% [8]. The sample size came out to be 110, with 55 participants in each group.

Participants in group A included those more than 18 years of age, both genders, admitted to the ICU and requiring prolonged intubation. Those patients with preoperatively planned tracheostomies were also included. Group B included patients with upper airway obstruction who presented with stridor. Patients with expiratory stridor and lower airway pathology were not included in the study.

Informed consent was obtained from all the patients or attendants prior to any intervention as part of an ethical concern. Patients or their attendants (in the case of unconscious or intubated patients) were informed about their inclusion in the study, the procedure they would undergo, its benefits, and the risks involved.

Patients were divided into two groups, i.e., elective tracheostomies and emergency tracheostomies. In group A, data regarding the frequency of complications of elective tracheostomies was noted, while in group B, that of emergency tracheostomies was noted. All patients were subjected to examinations and investigations pre- and postoperatively. X-ray chest, X-ray soft tissue neck anteroposterior (AP), and lateral view routine blood and urine examination, CT scan of head and neck, direct laryngoscopy, and biopsy (for laryngeal cancer patients) were done. Surgery was done by the final-year PGR or consultant. Patients were examined and followed up for three months for any complications.

Information concerning the patient's age, gender, indications, type of anesthesia, and complications was studied. All patients were followed for three months for adverse events related to the tracheostomy. Complications were divided into six main groups based on the literature review [5-7]. All information was collected on a Proforma.

Data were entered and analyzed in SPSS version 23. For qualitative variables like gender, indication, type of anesthesia, and complications, frequency was calculated. For quantitative variables like age, a mean with a standard deviation was calculated. A chi-square test was used to compare the complications between the two groups. A P-value of <0.05 was taken as significant.

## Results

We enrolled 110 patients in the current study, 55 (50%) in each group, with a mean age of 48.47±12.68 years in group A and 49.54±10.99 years in group B. The overall mean age of patients in the current study was 49.01±11.82 years. The total number of patients in different age groups is shown in Table 1.

**Table 1:** Age of patients in study groups A and B

Age groups	Study groups		Total N (%)
	Group A (elective tracheostomy) N (%)	Group B (emergency tracheostomy) N (%)	
<40 years	16 (29.1)	14 (25.5)	30 (27.3)
41–50 years	18 (32.7)	12 (21.8)	30 (27.3)
51–60 years	5 (9.1)	20 (36.4)	25 (22.7)
>61 years	16 (29.1)	9 (16.4)	25 (22.7)
Total	55 (100)	55 (100)	110 (100)
Mean age ± SD	48.47±12.68	49.54±10.99	49.01±11.82

In group A, 40 (72.7%) patients were male and 15 (27.3%) patients were female, and in group B, 37 (67.3%) patients were male and 18 (32.7%) patients were female, with a p-value (p=0.533), as shown in Table 2.

**Table 2:** Proportion of gender in study groups A and B

Gender	Study groups		Total N (%)	p-value
	Group A (elective tracheostomy) N (%)	Group B (emergency tracheostomy) N (%)		
Male	40 (72.7)	37 (67.3)	77 (70)	0.533
Female	15 (27.3)	18 (32.7)	33 (30)	
Total	55 (100)	55 (100)	110 (100)	

The results of postoperative complications in both groups A and B were observed as surgical emphysema (2 [3.6%] vs. 5 [9.1%] p=0.241), hemorrhage (2 [3.6%] vs. 4 [7.3%], p=0.401), wound infection (3 [5.5%] vs. 6 [10.9%], p=0.297), tube blockage (0 [0.0%] vs. 1 [1.8%], p=0.315), tube displacement (0 [0.0%] vs. 3 [5.5%], p=0.079), and tracheoesophageal fistula (0 [0.0%] vs. 2 [3.6%], p=0.154), as shown in Table 3. There was no significant difference between the two groups in terms of the type of complications.

**Table 3:** Incidence of complications in study groups A and B

Complications	Study groups				Total		P-value
	Group A (elective tracheostomy)		Group B (emergency tracheostomy)				
	Yes, N (%)	No, N (%)	Yes, N (%)	No, N (%)	Yes, N (%)	No, N (%)	
Surgical emphysema	2 (3.6)	53 (96.4)	5 (9.1)	50 (90.9)	7 (6.4)	103 (93.6)	0.241
Hemorrhage	2 (3.6)	53 (96.4)	4 (7.3)	51 (92.7)	6 (5.5)	104 (94.5)	0.401
Wound infection	3 (5.5)	52 (94.5)	6 (10.9)	49 (89.1)	9 (8.2)	101 (91.8)	0.297
Tube blockage	0 (0.0)	55 (100)	1 (1.8)	54 (98.2)	1 (0.9)	109 (99.1)	0.315
Tube displacement	0 (0.0)	55 (100)	3 (5.5)	52 (94.5)	3 (2.7)	107 (97.3)	0.079
Tracheoesophageal fistula	0 (0.0)	55 (100)	2 (3.6)	53 (96.4)	2 (1.8)	108 (98.2)	0.154

In the current study, we found that overall complications in elective tracheostomies were 7 (12.7%) and in emergency tracheostomies were 21 (38.2%). Complications were three times more common in emergency tracheostomies than elective tracheostomies.

## Discussion

Tracheostomies are one of the most frequent surgical procedures; however, they are not always without problems. In this research, 110 tracheostomies (55 [50%] elective and 55 [50%] emergency) were prospectively examined to determine their complications and to determine if there are any notable differences in terms of the incidence of complications between the two types of tracheostomies.

In a study by Choudhury et al. [8], the mean age was 40.46 years in the elective tracheostomy group and 50 years in the emergency tracheostomy group, with 55.66% of patients in the 31-50-year age group. In elective tracheostomy, 86.67% of patients were male, and in emergency tracheostomy, 90.0% of patients were male. The results of postoperative complications in both groups were observed, and the overall complications in electively done tracheostomies were 9.99%, while in tracheostomies done in emergencies, they were 33.35%. These results support the findings of the current study. In the study by Debesh Chandra Talukdar et al. [9], the prevalence of complications was consistent with our research findings.

Another study on elective tracheotomies revealed that problems occur between 8.94% and 19.64% of the time, respectively [10]. Analysis of prior research indicated that complications for emergency tracheostomies were 41%, 51%, and 58.62% [9]. In this instance, emergency tracheostomies resulted in problems three times more often than elective tracheostomies, which was similar to our study findings.

According to research by Anehosur et al. [11], emergency tracheostomies are more likely to result in complications than elective ones, just like our results.

Delany et al. [12] did a meta-analysis and found that the incidence of wound infection was 6.6%, hemorrhage was 5.7%, and the incidence of other major complications was 2.6%, with the overall incidence of complications being 14.9%, which was closer to our values.

Tracheostomy is a surgical procedure that may be used in emergency situations or for elective reasons. It is possible for this procedure to have a number of complications; however, the frequency and seriousness of these complications will depend on the interaction of the various contributing factors. Freburg-Hoffmeister et al. [13] reported hemorrhage as a major complication with an incidence of 3.4%, which was closer to our findings. Kligerman et al. [14] found the overall incidence of complications of tracheostomy to be 35.5%, which was not very different from our results. Akhlaq et al. [15], in their research done in Lahore, Pakistan, found that complications in the elective group were 20.59% and in the emergency group were 53.13%, which were different from our study. The prevalence of different early problems linked to the open surgical tracheostomy component in their setup was assessed and studied by Khammas et al. [16]. With a mean age of 46.85 years, there were 63.4% men and 36.6% women. Among the tracheostomies, around 56.25% were emergency tracheostomies, and 43.75% were elective. Of these, 54.46% were performed to relieve upper airway blockage. Early problems occurred more often (30.35%) in emergency tracheostomies than in elective procedures (8.92%); these results were not very different from our findings. In research [17], emergency tracheostomies are more likely to result in complications than elective ones.

Our study had a few limitations, i.e., the duration of the study was short, and it was based on participants from a single center with a limited sample size. We recommend a longer study with a larger sample size be conducted at multiple centers to address the complications more accurately, as these findings may improve the management of patients and also improve their lives.

## Conclusions

We found overall complications in elective tracheostomies at 12.7% and in emergency tracheostomies at 38.2%. Complications were three times more common in emergency tracheostomies than elective tracheostomies. Our discussion concluded that patients who underwent an emergency tracheostomy encountered a higher rate of postoperative complications as compared to those who had an elective tracheostomy.
